# Therapeutic impact of organism isolation in management of patients with pyogenic vertebral osteomyelitis

**DOI:** 10.1186/2193-1801-3-62

**Published:** 2014-02-01

**Authors:** Toshiya Tachibana, Tokuhide Moriyama, Keishi Maruo, Shinichi Inoue, Shinichi Yoshiya

**Affiliations:** Department of Orthopaedic Surgery, Hyogo College of Medicine, 1-1 Mukogawa-cho, Nishinomiya, Hyogo, 663-8501 Japan

**Keywords:** Vertebral osteomyelitis, Organisms, Biopsy, Antibiotic

## Abstract

In management of patients with pyogenic vertebral osteomyelitis, organism isolation by biopsy is generally considered to be of primary importance when constructing a treatment plan. In our clinical practice, however, patients can be successfully treated even without identifying the organisms. The objective of this study is to review our clinical experiences and clarify the therapeutic impact of organism isolation. Forty patients who were conservatively managed in our institution constituted the base of this study. The average follow-up period was 16.7 months. Among the study subjects, 13 patients underwent percutaneous needle biopsy and the organism was identified in 6 patients. Additionally, the organism was isolated from the sample obtained from blood and possible foci in 10 patients. In total, the causative organism was identified in 15 of the 40 patients (37.5%). Patients were divided into two groups based on whether the organism was identified by culture (Groups A and B, with and without organism isolation respectively). The duration of antibiotic therapy was not significantly different between the groups (Group A: 4.8 ± 1.6 months, Group B: 4.3 ± 2.1 months), while subsequent mortalities in Group A and B were 13.3% and 8% without significant intergroup difference. Organism isolation did not productively help select the effective antibiotics and reduce the treatment period or mortality rate in treatment of patients with pyogenic vertebral osteomyelitis. Therefore, current strategic antibiotic therapy may be effective in eradicating infection even without identification of the causative organism in treatment of patients with pyogenic vertebral osteomyelitis.

## Background

In management of pyogenic vertebral osteomyelitis (PVO), isolation of the causative organism is generally considered to be a key to constitution of an effective treatment regimen. Therefore, a culture of the sample obtained from the percutaneous spinal biopsy is primarily performed to find out the antibiotics to which the identified organism is sensitive (Currier et al. 2006; Grados et al. 2007; Mylona et al. 2008). In addition to the biopsied specimen, other samples from blood and other possible foci are also subjected to culturing to survey for the potential source of infection (Carragee 1997; Weinberg and Silber 2004). Regarding the significance of bacteriological examination of the biopsied specimen, it has been reported that the frequency of organism isolation by biopsy is consistently feasible and has a broad range in several studies (Currier et al. 2006; Hadjipavlou et al. 2000,2004; Lucio et al. 2000). If isolation of organisms is necessary for PVO treatments, then patients who did not have organisms isolated would have poor prognosis; however, in our clinical practice, patients with PVO can be successfully treated even without the identification of organisms. Consequently, the significance of percutaneous biopsy and acquisition of local tissue for a culture seem questionable. Therefore, the objective of this study is to review our clinical experiences and clarify the therapeutic impact of organism isolation on the treatment of patients with PVO.

## Methods

From 1996 to 2006, 68 patients with diagnosis of PVO were selected as the primary study cohort based on our clinical database. By reviewing the clinical record of each of these 68 patients, 40 patients who did not receive surgical treatment were identified and these patients constituted the base of this study population. Organism identification was attempted by culture of blood and samples obtained from the possible foci of infection in those patients, while 13 of the 40 patients underwent percutaneous needle biopsy.

Based on the culture results, the included patients were divided into two groups: Group A (15 patients), causative organisms were isolated; and Group B (25 patients), organisms were not identified. The mean age for Group A was 62.1 years (range, 29–77 years), and the follow-up period averaged 12.8 months (range, 2–54 months). In this group of patients, the causative organism and the source of the specimens revealing positive culture were reviewed in the patient record. In the remaining 25 Group B patients, the mean age and follow-up period were 64.3 years (range, 15 – 84 years) and 64.3 months (range, 2–91 months) respectively.

All the patients gave the informed consent prior being included into the study. The study was authorized by the local ethical committee, Hyogo College of Medicine and was performed in accordance with the Ethical standards of the 1964 Declaration of Helsinki as revised in 2000.

Based on the review of patient records, clinical parameters such as affected spinal level, comorbidity, selection of antibiotics, duration of antibiotic administration required for subsidence of infection, and subsequent mortality were compared between the groups.

In the statistical analysis, t-tests were performed with Excel (Microsoft Corporation, Redmond, WA, USA) and a Fischer’s exact test was performed using SPSS (SPSS, Chicago, IL, USA). A p-value of < 0.05 was considered to indicate significance.

## Results

Among the 13 patients who underwent percutaneous needle biopsy, organisms were identified in 7 patients (53.8%). The organisms isolated from the spinal biopsy samples are shown in Table 1. Potentially causative organisms were isolated from blood and other foci in 10 patients as shown in Table 2. In total, organisms were identified in 15 patients (Group A); however, the antibiotics that were already administered at the corresponding time period were found to be effective in 14 of these 15 patients (93%). In other words, isolation of the organism induced a change in antibiotic selection in only one of the 13 patients with positive culture results in needle biopsy. Therefore, identification of the organism could not provide a therapeutic benefit in a majority of the patients. The total number of antibiotics given is shown in Table 3. Antibiotic administration was start with use of a single agent, while change to other agents or use of multiple agents were considered depending on sequential change in inflammatory reaction. In Group A, There were cervical lesion in one patient, thoracic lesions in 7 patients, and lumbar lesions in 7 patients; while, In Group B, there were cervical lesions in 3 patients, thoracic in 7 patients, and lumber lesions in 18 patients. Comorbidities were malignant diseases in 5 patients, diabetes in 3 patients, autoimmune diseases in 2 patients, and other infectious lesions in 4 patients in Group A; while for Group B there were malignant diseases in 3 patients, diabetes in 9 patients, autoimmune diseases in 3 patients, and other infectious lesions in 4 patients. There were no significant differences between groups.

**Table 1:** **Organisms isolated from the spinal lesion**

Organisms	
MSSA	2 cases
*Streptococci*	2 cases
*Pseudomonas aeruginosa*	2 cases
Others	1 case
Total	7 cases

MSSA: *Methicillin sensitive staphylococcus aureus*.

**Table 2:** **Organisms isolated from blood or foci in other regions**

Organisms	
MRSA	4 cases
MSSA	1 case
*Streptococci*	2 cases
*Pseudomonas aeruginosa*	3 cases
Total	10 cases

MRSA: Methicillin resistant staphylococcus aureus, MSSA: Methicillin sensitive staphylococcus aureus.

**Table 3:** **Total number of antibiotics administered during the treatment period**

	A (with isolation of organism)	B (without isolation of organism)
Cephalospolins	8	11
Extended spectrum penicillins		
Plus beta lactamase inhibitor	5	6
New quinolones	5	6
Carbapenems	4	2
Glycopeptides	3	0
Aminogricosides	0	2
Tetracyclines	1	2
Others	2	3

The duration of antibiotic administration required for subsidence of infection was not significantly different between the groups (4.8 ± 1.6 months in Group A and 4.3 ± 2.1 months in Group B; Table 4). Subsequent mortalities were 13.3% and 8.0% in Groups A and B respectively with no intergroup difference (Table 5).

**Table 4:** **Duration of antibiotic therapy**

	Duration of antibiotic therapy (months)
A (with isolation of organism)	4.8 ± 1.6
B (without isolation of organism)	4.3 ± 2.1

No significant difference between the groups.

**Table 5:** **Mortality of the patients**

	Survivors (n)	Non-survivors (n)	Mortality rate
A (with isolation of organism)	13	2	13.3%
B (without isolation of organism)	23	2	8.0%

No significant difference between the groups.

## Discussion

In constituting a management plan for patients with PVO, in general, a blood culture is initially performed because the infection may be hematogenous in origin (Currier et al. 2006; Grados et al. 2007). In the present study, other infectious lesions such as pyogenic arthritis were concomitantly present in 7 patients. In the other 33 patients, the original focus of infection was unknown; however organisms may have been known to spread to the spine hematogenously. When the blood culture fails to reveal the potentially causative organisms, percutaneous biopsy can be the next option to survey for this. In previous clinical studies, the positive rate in a blood culture performed for patients with PVO ranged from 55.6% to 59% (Carragee 1997; Weinberg and Silber 2004). In the present study, however, culture of blood and samples obtained from possible foci demonstrated positive results in only 10 patients (25%). By contrast, a culture of the needle biopsy sample showed positive results in 53.8% of the patients. The rates of positive culture results from needle biopsy have been reported with variable values ranging from 30.4% to 86% (Currier et al. 2006; Hadjipavlou et al. 2000; Sehn and Gilula 2010), and the rates of sample acquisition by transpedicular discectomy have been reported as well (range, 73.5% to 78%) (Hadjipavlou et al. 2004; Lucio et al. 2000). Therefore, 14% to 69.6% of patients with PVO were treated without isolating the causative organisms.

There has been only one study investigating the effect of organism isolation on the enhancement of treatment efficacy. In this report, Rankine et al. demonstrated that the results of biopsy lead to change in management in 7 out of 20 patients (35%) (Rankine et al. 2004). In the present study, isolation of the organism induced a change in antibiotic selection for only 1 of the 13 patients with positive culture results in needle biopsy, while isolation of the organism from blood or other foci did not lead to a change in antibiotic selection in any of the patients.

As for the duration of antibiotic therapy for patients with PVO, administration for a relatively long time period is generally recommended. Early cessation of antibiotics at 4 to 6 weeks has been reported to be associated with a higher recurrence rate (Currier et al. 2006; Sapico and Montgomerie 1979; Roblot et al. 2007), and thus administration for more than 12 weeks is the general consensus (Grados et al. 2007). The average time period of antibiotic therapy (4.5 months) in this study corresponds to the duration reported in previous studies. Regarding whether isolation of organisms can help construct an effective treatment protocol and reduce the treatment period, the results of this study showed no difference in duration of antibiotic therapy regardless of the status of organism identification. The mortality rate in patients with PVO has been reported to be relatively low ranging from less than 5% to 16% (Currier et al. 2006). The mortality rates in the two groups of this study (8% in Group A and 13.3% in Group B) correspond to those in previous studies, and isolation of the organisms did not influence the mortality rate. Comorbidities including malignant disease which may influence the mortality rate were also not different between groups. There has been no study investigating the significance of percutaneous needle biopsy in treatment of patients with PVO by comparing the outcome of the patient populations with and without isolation of the organism. In regards to the selection of antibiotics, since *methicillin sensitive staphylococcus aureus* (MSSA) used to be the most frequently identified organism, the agent targeting this species such as cefazolin was conventionally selected as an initial choice (Bhavan et al. 2010; Nather et al. 2005). However, since *methicillin resistant staphylococcus aureus* (MRSA) has emerged as a predominant organism causing PVO, anti-MRSA antibiotics may be currently added as an option before isolation of the organism (Bhavan et al. 2010). If these agents cannot control the infection, the switching of antibiotics to those with a broad spectrum and effectiveness against gram negative organisms can be considered (Weinberg and Silber 2004). It is thought that this concept of current antibiotic selection can effectively cope with PVO regardless if the causative organism is isolated.

This study has several limitations. First, the study was based on a retrospective chart review and was not prospectively designed. Therefore, treatment decision making (indication of percutaneous biopsy as well as antibiotic selection and duration of administration) was arbitrarily determined by the responsible surgeon. Moreover, the sample size was relatively small and the follow-up period in some patients may not have been long enough to investigate the clinical outcome. Moreover, in the general population, pathogen distribution may be different from the patient population included in this study. A prospectively designed, multi-center clinical trial with consistent treatment criteria is required to draw validated conclusions.

## Conclusions

The present study has shown that organism isolation did not productively help select the effective antibiotics and reduce the treatment period or mortality rate in the treatment of patients with PVO. Although the study results do not contradict the clinical significance of needle biopsy for this patient population, the current strategic antibiotic therapy may be effective in eradicating infection even without identifying the causative organism in the treatment of patients with PVO.
